# Learning effectiveness of simulation based teaching in Gynaecology and Obstetrics among medical students: a mixed-methods study

**DOI:** 10.3389/fmed.2025.1652105

**Published:** 2025-08-25

**Authors:** Shamaila Mohsin, Brekhna Jamil, Kiran Imtiaz Khan, Safiullah Virk, Abdul Momin Rizwan Ahmad

**Affiliations:** ^1^Department of Public Health, Armed Forces Post Graduate Medical Institute (AFPGMI), National University of Medical Sciences (NUMS), Rawalpindi, Pakistan; ^2^Institute of Health Professions Education & Research, Khyber Medical University (KMU), Peshawar, Pakistan; ^3^Department of Paediatric Dentistry, Army Medical College, National University of Medical Sciences (NUMS), Rawalpindi, Pakistan; ^4^Army Medical College, National University of Medical Sciences (NUMS), Rawalpindi, Pakistan; ^5^Department of Health Sciences, University of York, York, United Kingdom; ^6^Department of Human Nutrition and Dietetics, NUST School of Health Sciences, National University of Sciences & Technology (NUST), Sector H-12, Islamabad, Pakistan

**Keywords:** learning, effectiveness, simulation, Gynaecology, Obstetrics, medical students

## Abstract

**Introduction:**

Simulation-Based Teaching (SBT) has emerged as an educational strategy to enhance clinical competence among medical students, particularly in Gynaecology and Obstetrics.

**Objective:**

This study assessed the effectiveness of learning SBT and explored the enablers and challenges associated with implementing SBT in Gynaecology and Obstetrics.

**Methods:**

Using a sequential explanatory mixed methods approach a study was conducted in Skills Lab at Pakistan Emirates Military Hospital (PEMH), Rawalpindi during May–Sep 2024. A pre/post design study was conducted on final-year medical students using a validated questionnaire Simulation Learning Effectiveness Inventory (SLEI-SCM). The qualitative component explored students’ and faculty perception of SBT through In-depth interviews. The quantitative data was analyzed using SPSS (V.25) while the qualitative data was conducted through Braun and Clarke Thematic framework.

**Results:**

A total of 190 participants participated in the quantitative study. The pre-training mean score of the participants 89.4 significantly improved to 139.5 post-training (Mean Difference = 50.1). Post-intervention results revealed significant improvements (*p* < 0.001) in learning effectiveness such as workshop content and resource availability. The in-depth interviews conducted with 23 participants, students and facilitators, revealed several key themes.

**Conclusion:**

SBT significantly enhanced undergraduate learning outcomes in Gynecology and Obstetrics, in workshop content, resource availability, and clinical confidence.

## 1 Introduction

Maternal mortality and morbidity rates in low- and middle-income countries (LMICs) persist at alarming levels, despite global efforts to improve maternal healthcare outcomes (1). Evidence indicates that Obstetric emergencies are a leading cause of maternal mortality, often exacerbated by deficiency in clinical skills and teamwork among healthcare providers (2). Simulation-based teaching (SBT) in Gynaecology and Obstetrics has emerged as an effective educational strategy to address these challenges and enhance the preparedness of healthcare professionals, particularly undergraduate medical students (3). Studies indicate that simulation-based training in Gynaecology and Obstetrics has been shown to have a positive impact on the acquisition of clinical skills among medical students (4, 5). In a study conducted by Everett et al. it was found that simulation training reduced clinical stress and increased clinical satisfaction in students (6). Similarly, Fransen et al. reported that high-fidelity simulation improved students’ understanding of obstetric procedures and increased their self-assurance (7). Gorantla et al. noted that Gynaecologic simulation training increased students’ confidence in performing procedures and their interest in women’s health (8). Hafeez et al. further supported these findings, showing that simulation training improved surgical performance by Gynaecology and Obstetrics trainees (9). These studies collectively suggest that simulation-based training is effective in enhancing clinical skills and confidence in Gynaecology and Obstetrics among medical students. Evidence also suggests that incorporation of new technologies and advancements in simulation-based training enhances the educational experience for healthcare providers (10). This includes the utilization of virtual reality simulations, augmented reality tools, and advanced medical manikins that can provide a more realistic training environment (11).

However, while SBT has been widely adopted in high-income countries and shown to improve technical skills and clinical outcomes, its application and effectiveness in LMICs remain relatively unexplored (12). LMICs face unique barriers to healthcare delivery, including limited resources, infrastructure constraints, and variations in healthcare systems, which may impact the implementation and effectiveness of SBT initiatives in these settings (13, 14). The research gap also lies in the scarcity of evidence specifically examining the effectiveness of SBT tailored to the needs and contexts of undergraduate medical education in LMICs (15). Existing studies predominantly originate from high-income countries, limiting their generalizability to LMIC settings. Consequently, there is a critical need for rigorous research that investigates the effectiveness of SBT in Gynaecology and Obstetrics for undergraduate medical students in LMICs (16). There is a need for more studies on the long-term retention of skills learned through simulation-based training in Obstetrics (17). By addressing these gaps, we can enhance the quality of education and ultimately improve patient outcomes in Obstetrics and Gynaecology. Research on the use of simulation-based teaching in Pakistan has been limited in one such study in Pakistan among Gynaecology residents indicated a very high satisfaction level was attained after simulation-based teaching (18). Evidence reiterates that Simulation-based teaching offers a safe environment for medical students to practice and refine these skills without risk to real patients (7, 18). This study aims analyze the effectiveness of simulation-based training in enhancing the learning experience of medical students in Gynaecology and Obstetrics and understand the perceived barriers and facilitators influencing simulation-based training in the learning of clinical skills among medical students as a proof-of-concept study.

## 2 Materials and methods

### 2.1 Study design

This study employed a sequential explanatory mixed methods approach, combining quantitative and qualitative data collection and analysis to investigate the effectiveness of simulation-based training (SBT) in Gynecology and Obstetrics for undergraduate medical students.

### 2.2 Phase one: Quantitative study

In the first phase of the study the quantitative data was obtained through a pre and post intervention design from participants of the simulation session. The simulation-based workshop was a mandatory component of the final-year undergraduate curriculum in Obstetrics and Gynaecology at Army Medical College, in collaboration with the Department of Gynaecology and Obstetrics at Pak Emirates Military Hospital (PEMH). As part of their clinical rotation, all final-year MBBS students (*n* = 201) were required to attend the workshop during their scheduled posting. Inclusion criteria included those under-graduate medical students of final year who had completed or were currently participating in simulation-based training sessions related to Gynaecology and Obstetrics. Students who were unable or unwilling to participate in the research study due to personal or scheduling conflicts were excluded. A convenience sampling strategy to select students based on their exposure to SBT in Gynaecology and Obstetrics was employed. Data was collected by Simulation Learning Effectiveness Inventory (SLEI-SCM; 32). The SLEI-SCM evaluates six domains of Simulation-Based Training (SBT) learning effectiveness through three subscales. These domains encompass course arrangement, equipment resources, debriefing, clinical abilities, problem-solving, and confidence. It consists of 31 items. Responses were recorded on a Likert-type 5-point scale, ranging from 1 (strongly disagree) to 5 (strongly agree). The instrument’s total score ranges from 31 to 155, with higher scores indicating greater learning effectiveness. The SLEI-SCM has demonstrated reliability and validity, with a Cronbach’s *α* coefficient of 0.95 and a 2-week test–retest reliability of 0.88 (32). Prior to actual data collection, a pilot test of the questionnaire was conducted to assess the questions’ quality, strengths, and weaknesses.

The participants were invited to participate through announcements during the simulation session and their willingness to participate voluntarily. The demographic information sheet and the questionnaire were distributed to participants after the SBT session in the skills laboratory. Participants were provided with the questionnaire in the printed format. They were instructed to complete the questionnaire independently and return it within a specified timeframe (15–20 Min). Completed forms were returned anonymously.

### 2.3 Simulation workshop

A 2 h workshop consisted of half-hour lecture along with case scenarios and one and half hour hands on training on handling of instruments, safety practices, skills to conduct delivery on a mannequin. These scenarios were standardized and based on current clinical guidelines in the undergraduate curriculum. The simulation scenarios and workshop content were collaboratively designed by HoD Gynaecology and three consultant faculty members from the Department of Gynaecology and Obstetrics at PEMH. The development process included: identifying core competencies required for undergraduate obstetric training and mapping learning objectives to simulation activities. These scenarios were reviewed and approved by the Department of Medical Education (DME) at Army Medical College, ensuring both academic rigor and pedagogical validity. Instructors of the simulation sessions were second year residents and consultant faculty members trained in simulation-based medical education. Prior to workshop delivery, they participated in a 1 day workshop conducted by DME. The workshop was conducted in the simulation laboratory at PEMH for every batch (20–25) students of final year students. During the workshop participants were subdivided into four equal teams, each comprising of 4–5 students, mirroring real-life labor room scenarios. The students received traditional lecture-based instruction for half an hour via PowerPoint followed by hands-on practice on a simulator. Preparatory reading materials was emailed one week beforehand. The simulation sessions utilized the Mama Natalie birthing simulator. This mannequin allows for realistic practice of vaginal delivery techniques, management of postpartum hemorrhage, neonatal resuscitation, uterine massage and fundal assessment.

### 2.4 Quantitative data analysis

Participant demographic data, such as age, gender, single-child family status, appointment holder status (class leader), personality type, and personal motivation to pursue a medical career, was also collected. The quantitative data was analyzed using SPSS version 25.0 software (SPSS, Inc., Chicago, IL, United States). Descriptive analysis was conducted to examine the SLEI-SCM scores and participant characteristics with means (± SD) and frequency distributions. Paired t-test was used. Missing data was addressed through mean value substitution, and statistical significance would be set at *p* < 0.05. Informed consent was obtained from all study participants. Confidentiality and anonymity of participants was ensured throughout the study.

### 2.5 Phase two: Qualitative study

Study participants who completed the questionnaire as well as facilitators of the simulation session were recruited through the “opt-in” approach to participate in in depth interviews. These interviews were semi-structured to allow for consistency through guiding questions, while also offering flexibility to explore emerging themes and individual reflections in greater depth. This approach was considered suitable for our study as our aim was to delve into perspectives and gain detailed insights into learner and facilitator’s experience. An interview guide was developed after a thorough review of existing literature related to simulation-based training in Gynaecology and Obstetrics by the lead researcher and the supervisor. Key concepts influencing the learning experience of medical students such as perceptions of Simulation-Based Training (SBT), impact on Learning Outcomes, challenges, instructor support, application to Clinical Practice were explored. A pilot test of the interview guide was conducted to assess its clarity, comprehensiveness, and appropriateness. The guide was revised based on feedback from the pilot test.

### 2.6 Qualitative data analysis

Data analysis was done by the Six Step Braun and Clarke Thematic Framework (19). The process of collecting data and analysis was carried out simultaneously for the study. Firstly, two researchers (SV and KK) cleaned the transcripts meticulously and read and re-read them in order to familiarize themselves. Secondly initial codes were generated from the transcripts. Thirdly two researchers (SM and BJ) generated sub-theme from the codes and then subsequently generated themes. In the fourth stage the researchers met with the supervisor (BJ) to refine the themes. Finally verbatim was again analyzed to identify key quotes that would supplement the themes related to the effectiveness of SBT in enhancing technical skills among undergraduate medical students.

## 3 Results

### 3.1 Quantitative phase

#### 3.1.1 Demographic characteristics

Out of a total cohort of 201 final-year students, 190 students participated in the study, resulting in a participation rate of 94.3%. The demographic characteristics revealed that the study sample had a mean age of 22.9 years (SD ± 1.1). The study participants were predominantly male (61.6%) while almost one fourth (38.4%) of the participants were females as shown in Table 1. Most participants were born in urban areas (75.3%) while a proportion (24.7%) of the students were born in rural areas as shown in Table 1. The majority (64.2%) of the study participants came from nuclear families whereas a proportion (24.2%) were from joint families as shown in Table 1.

**Table 1:** Sociodemographic characteristics of participants.

S. No	Variables	Frequency	Percentage
1.	Age		
	Mean ± SD	22.9 years (SD ± 1.1)	
2.	Gender		
	Male	117	61.6%
	Female	73	38.4%
3.	Birth place		
	Urban	143	75.3%
	Rural	47	24.7%
4.	Family type		
	Joint	46	24.2%
	Nuclear	122	64.2%
	Mixed	22	11.6%

SD = Standard Deviation.

#### 3.1.2 Learning effectiveness of the simulation workshop

**Figure 1:**
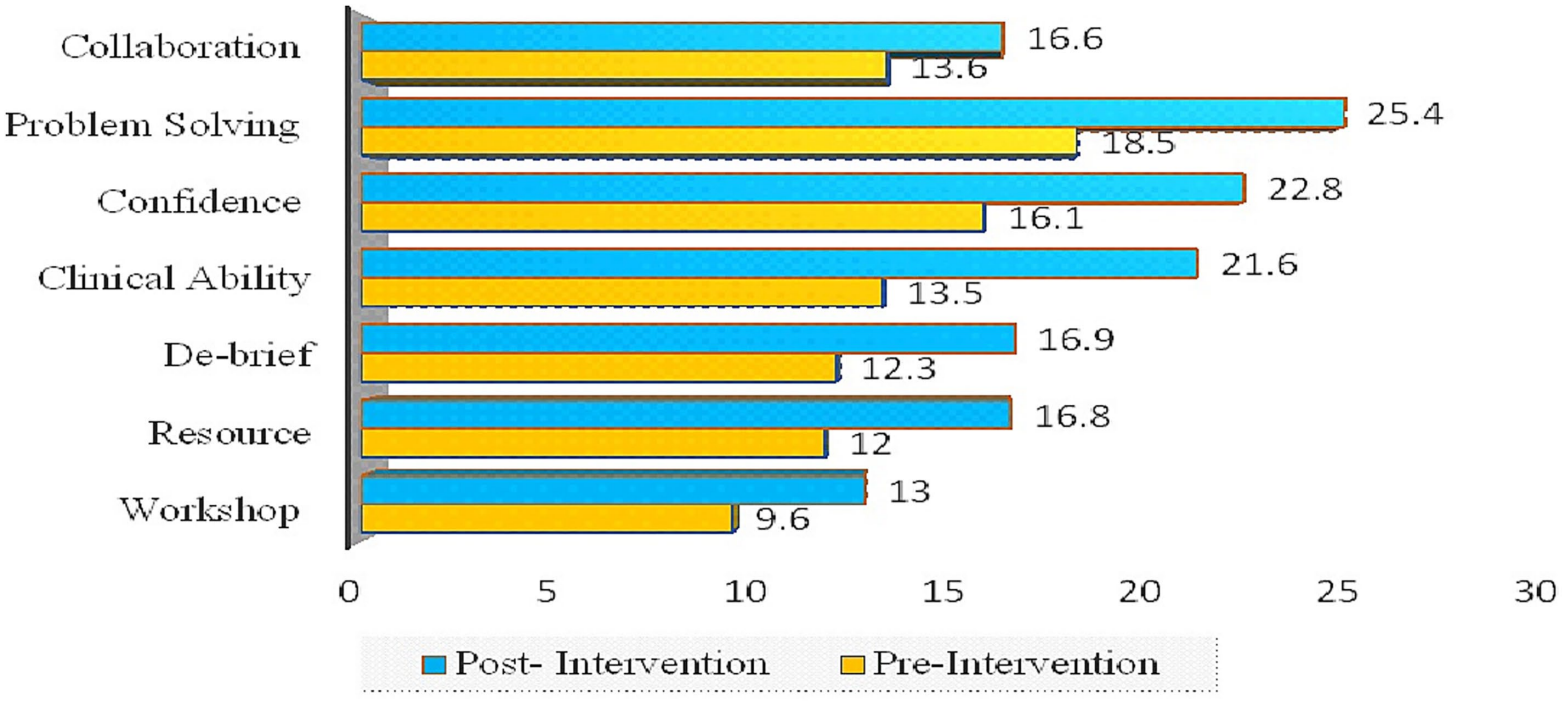
Comparison of mean scores on domains of learning effectiveness.

The intervention’s effectiveness was evaluated by measuring participant responses across seven domains: Workshop Content, Resource Availability, Debrief Quality, Clinical Ability, Confidence, Problem Solving, and Collaboration. The mean scores of seven domains of Learning Effectiveness before and after an intervention were compared. The Workshop domain revealed that the mean score increased from pre-intervention 13 (SD = 2.3) to post-intervention 9.97 (SD = 2.1). Similarly, the Resource domain revealed that the mean score rose from 12 (SD = 2.4) to 16.8 (SD = 2.9) post-intervention as shown in Figure 1.

The results of the student’s paired t-test reveal statistically significant improvements across majority of the measured domains following the intervention. For the Workshop category, the t-value was 15.7, with a 95% confidence interval (CI: 2.7–3.4) (*p* < 0.00). Similarly, for Resource, the t-value was 18.1, with a (CI: 4.2–5.3) (*p* < 0.00). In the Debrief category, the t-value reached 16.7 (CI 4.0–5.1) (*p* = 0.11). Clinical Ability showed the largest effect, with a t-value of 24.6 and a (CI: 7.4–8.7) (*p* < 0.00) shown in Table 2. For Confidence, the t-value was 16 (CI: 4.3–5.5) (*p* < 0.00), while Problem Solving had a t-value of 16.5 (CI: 5.3–6.7) (*p* = 0.07). Lastly, in the Collaboration category, the t-value was 10.7 (CI: 2.5–3.6) (*p* < 0.00) shown in Table 2.

**Table 2:** Student’s paired t-test to compare scores in pre- and post-intervention.

Items	Paired t-test for equality of means				
			95% confidence interval of the difference		
	*t*	df	Upper	Lower	*p*-value
Workshop	15.7	**186**	2.7	3.4	**<0.001**
Resource	18.1	186	4.2	5.3	<0.001
Debrief	16.7	186	4.0	5.1	0.11
Clinical ability	24.6	186	7.4	8.7	<0.001
Confidence	16	186	4.3	5.5	<0.001
Problem solving	16.5	186	5.3	6.7	0.07
Collaboration	10.7	186	2.5	3.6	<0.001

Statistically significant differences at *p* < 0.05 (df = degrees of freedom).

The results of the student’s paired t-test (6.7) with a 95% confidence interval (CI: 13.6–7.4) reveal statistically significant (*p* < 0.00) improvement in learning effectiveness after the intervention shown in Table 3.

**Table 3:** Student’s paired t-test of total scores in pre- and post- intervention.

Item				95% confidence interval of the difference		
	Mean	t	df	Upper	Lower	*P*-value
Pre-workshop	89.4	6.7	**190**	13.6	7.4	**< 0.001**
Post-workshop	139.5					

Statistically significant differences at *p* < 0.05 (df = degrees of freedom).

The Pre-Training Mean Score of the participants was 89.4, which increased to 139.5, post training demonstrated a significant improvement. The mean difference calculated was 50.1, whereas as a high learning gain ratio (56%) suggests that the training resulted in improvement relative to the pre-training score as shown in Table 4.

**Table 4:** Learning gain.

Variable	Pre-training mean score	Post-training mean score	Mean difference (learning gain)	Learning gain ratio
Learning effectiveness	89.4	139.5	50.1	56%

### 3.2 Qualitative phase

A total of 23 interviews were conducted with students (*n* = 12) and faculty (*n* = 11) till data saturation was reached. The students’ ages ranged from 22 to 24 years, with a gender distribution of 60% female (*n* = 7) and 40% male (*n* = 5). The students came from a variety of urban and rural backgrounds. The characteristics of the students are detailed in Table 5.

**Table 5:** Sociodemographic characteristic of students.

Faculty reference no	Age	Gender	Education	Place of residence	Marital status
IDI-MS-01	23	F	Final year MBBS	Urban	Un-married
IDI-MS-02	22	F	Final year MBBS	Urban	Un-married
IDI-MS-03	22	F	Final year MBBS	Urban	Un-Married
IDI-MS-04	22	F	Final year MBBS	Rural	Un-married
IDI-MS-05	24	F	Final year MBBS	Urban	Un-married
IDI-MS-06	23	F	Final year MBBS	Urban	Un-married
IDI-MS-07	23	F	Final year MBBS	Rural	Un-married
IDI-MS-08	23	M	Final year MBBS	Urban	Un-married
IDI-MS-09	23	M	Final year MBBS	Urban	Un-married
IDI-MS-10	24	M	Final year MBBS	Urban	Un-married
IDI-MS-11	24	M	Final year MBBS	Rural	Un-married
IDI-MS-12	23	M	Final year MBBS	Urban	Un-married

Eleven faculty were interviewed till saturation was reached. This included seven residents and four consultants. The age of the participants ranged from 27 to 45 years. All the faculty members were female (*n* = 11). The residents were mostly third year trainees (*n* = 5) while the rest were second year residents (*n* = 2), while the consultants had more extensive experience 17.75 years (SD + 2) and contributed significantly to both clinical and academic roles. The characteristics of the faculty are detailed in Table 6.

**Table 6:** Socio-demographic characteristics of faculty (*n* = 11).

Faculty reference no	Age	Gender	Education	Work experience	Marital status
IDI-FM-01	27	F	FCPS-1	3 years	Married
IDI-FM-02	29	F	FCPS-1	3 years	Married
IDI-FM-03	25	F	FCPS-1	2 years	Married
IDI-FM-04	29	F	FCPS-1	2 years	Married
IDI-FM-05	28	F	FCPS-1	3 years	Married
IDI-FM-06	26	F	FCPS-1	3 years	Married
IDI-FM-07	28	F	FCPS-1	3 years	Married
IDI-FM-08	44	F	FCPS, CHPE	19 years	Married
IDI-FM-09	39	F	FCPS	15 years	Married
IDI-FM-10	43	F	FCPS, MHPE	18 years	Married
IDI-FM-11	45	F	FCPS	19 years	Married

*FCPS = Fellow of the College of Physicians and Surgeons, CHPE = Certificate in Health Professions Education. Statistically significant differences at *p* < 0.05 (df = degrees of freedom).

**Figure 2:**
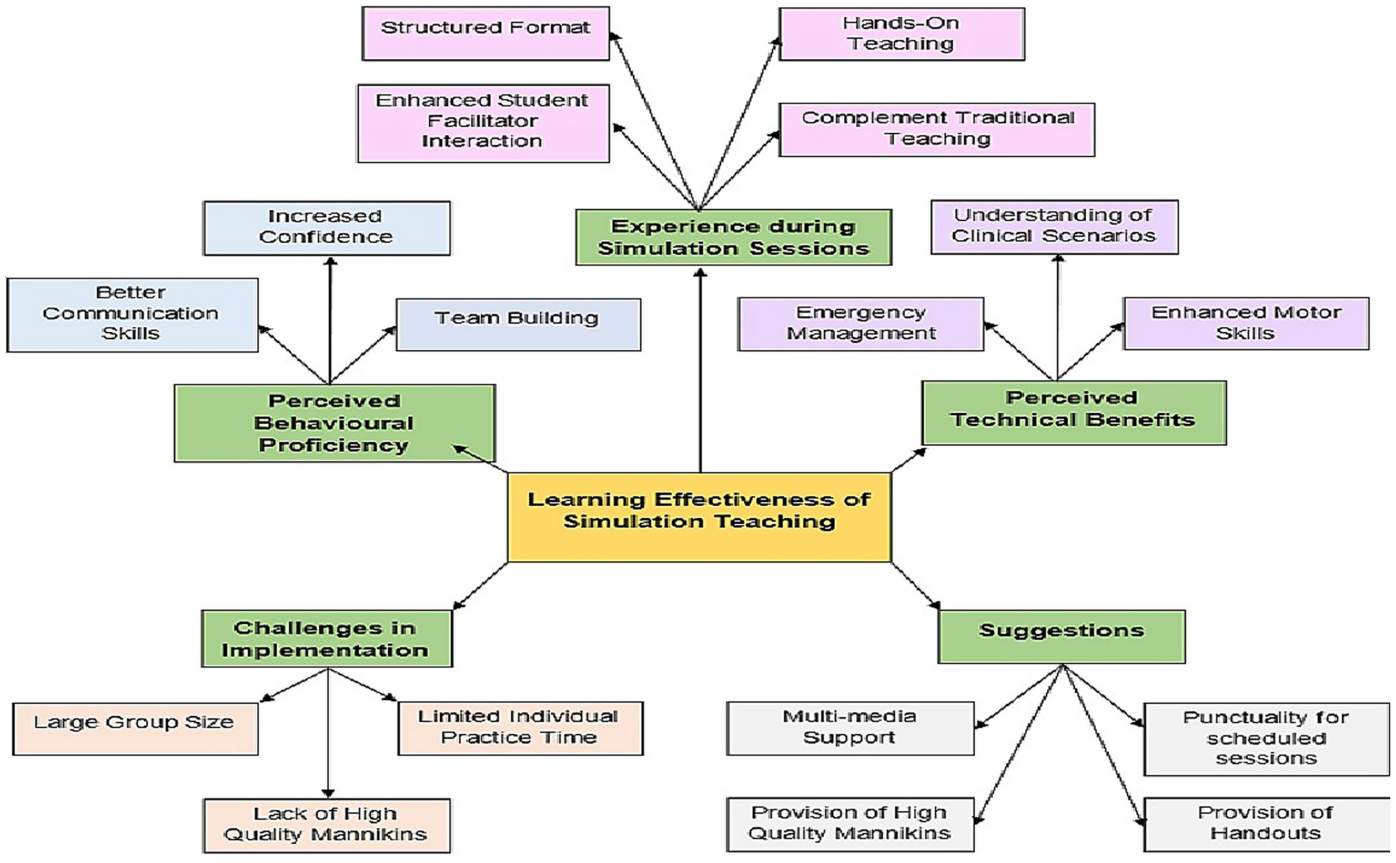
Concept map.

The qualitative research on simulation training in Gynae and Obstetrics revealed five key themes that encapsulate the participants’ insights and experiences; Experience during Simulation Sessions, Perceived Technical Proficiency, Perceived Behavioral Benefits, Challenges in Implementation, Suggestions as shown in Figure 2.

#### 3.2.1 Experience during simulation sessions

In qualitative interviews conducted with students and faculty Experience During Simulation Sessions including structured format, hands-on teaching, enhanced student facilitator interaction, complement traditional teaching.

##### 3.2.1.1 Structured format

Both students and faculty emphasized the importance of a structured format provided in the simulation sessions. The students revealed that during their simulation workshop was very well organized that allowed the learners to engage in a clear, step-by-step manner. One student noted, “*The simulation session was very structured. It (workshop) followed a clear form, which helped me to know what to expect and what is expected of me as the reading material and LOs (Learning Objectives) were shared one week before on the email. Our clinical coordinator gave us a proper schedule (Female Student, 24 yrs)”.*

##### 3.2.1.2 Hands-on teaching

Hands-on learning was repeatedly highlighted as a major advantage of simulation training. The students appreciated the opportunity to apply theoretical knowledge in a controlled, practical environment, which reinforced their learning and improved their technical skills. A student described the experience as follows: “*Usually, in the session at first, the teacher briefed us for about 15 min, then each student is instructed to basically…. perform a skill. So, after the session after each student has performed, the teacher is basically right there observing us. So, if any student goes wrong teacher corrects us right at the moment and tells us how we have to do this (Female Student, 23 yrs)”.*

##### 3.2.1.3 Enhanced student-facilitator interaction

Another key sub theme was enhanced interaction between students and facilitators during simulation sessions. The smaller group sizes and focused environment were seen as conducive to more meaningful engagement and personalized feedback. Faculty also valued this aspect, with a facilitator commenting, “*In these (simulation) sessions they with us for almost two hours we get to know them. This allows us to provide more individualized attention and conduct our teaching to the needs of each student if they have a query I answer there and then (Resident, 27 years)”.*

##### 3.2.1.4 Complements traditional teaching

While simulation was highly valued, both students and faculty agreed that it serves as a complement to, rather than a replacement for traditional teaching methods. Simulation sessions were seen as an effective way to bridge the gap between theoretical knowledge and practical application. Faculty supported this view, with one instructor stating, *“Simulation is an excellent adjunct to traditional teaching. It allows students to apply what they have learned in a safe environment, where they can make mistakes and learn from them without real-world consequences (Consultant, 44 years)”.*

#### 3.2.2 Perceived technical proficiency

The students and faculty highlighted several subthemes enhanced motor skills, emergency management, and understanding of clinical scenarios in this theme.

##### 3.2.2.1 Enhanced motor skills

Simulation training was found to significantly enhance motor skills, a critical aspect of technical proficiency in Gynecology and Obstetrics as both students and faculty emphasized that simulated environment allowed learners to refine their dexterity and precision in performing various procedures. One student shared, *“Before the simulation sessions, I struggled with certain procedures like episiotomy…. But after practicing with them, my hands just seemed to know what to do. My movements became more confident and precise (Female student, 24 years)”.*

##### 3.2.2.2 Emergency management

Simulation training was perceived to be highly valued for its role in preparing students for high-pressure situations, where quick thinking and swift action are essential. A student described the experience as follows: *“The workshops are intense, but that’s what makes them good. They (faculty) put you in scenarios such as in obstructed labor …where you have to act fast, just like in a real emergency. It’s helped me feel more prepared and less panicky when something goes wrong in an emergency (Female Student, 23 years)”*.

##### 3.2.2.3 Understanding of clinical scenarios

Another significant subtheme was the deepened understanding of clinical scenarios that students gained through simulation training. One student reflected, *“It’s one thing to know the steps in managing a case; it’s another to actually go through the process, make decisions, and see the outcomes in real-time (Male Student, 24 years)”*.

#### 3.2.3 Perceived behavioral benefits

The study revealed key subthemes under the central theme of perceived behavioral benefits, increased confidence, better communication skills, and team building.

##### 3.2.3.1 Increased confidence

One of the most significant behavioral benefits noted by both students and faculty was the increase in confidence resulting from simulation training to build self-assurance in their abilities to handle real-life clinical situations. A student described the experience, saying, *“Before the simulations, I was always second-guessing myself, especially in critical situations. But after working through different scenarios…, I have now started to trust my instincts. Now, I feel much more confident when I’m in the actual labor room rotation (Female Student, 23 years)”*.

##### 3.2.3.2 Better communication skills

Simulation training was also found to enhance communication skills among students, particularly in the context of patient care and teamwork. The realistic scenarios often require students to communicate clearly and effectively with both patients and colleagues, mirroring the demands of real clinical environments. One student shared, “*It really pushed me to improve my communication skills… whether I was explaining a procedure to my friend in the team or trying to act it out, I’ve learned how important it is to be clear the clarity should be there (Female Student, 23 years)”*.

##### 3.2.3.3 Team building

Teamwork is an essential component of healthcare and collaborative nature of the simulations encouraged students to work together, fostering a sense of camaraderie and mutual support. One student reflected on this aspect, saying, *“The team-based scenarios in the simulations were invaluable. They taught me how to rely on my colleagues and also how to contribute effectively to the team. We learned to trust each other…. which is something you do not get from traditional lectures”*.

#### 3.2.4 Challenges in implementation

Challenges in Implementation encompasses three significant subthemes: large group size, limited individual practice time, and lack of high-quality manikins.

##### 3.2.4.1 Large group size

One of the primary challenges identified was the large group size during simulation sessions. Both students and faculty noted that large groups made it difficult to ensure that each student had sufficient opportunities to participate actively in the simulations. A student expressed frustration, stating, *“When there are too many of us in a session, it becomes hard to fully learn…. You end up watching others more than practicing yourself, and that takes away from experience that’s so valuable in learning (Female Student, 24 years)”*.

##### 3.2.4.2 Limited individual practice time

Another significant challenge highlighted was the limited individual practice time available during simulation sessions. With large groups and time constraints, students often found it difficult to gain adequate hands-on experience, which is crucial for developing practical skills. A student shared their concern, saying, *“We get so little time to actually practice…. By the time it’s your turn, the session is almost over, and you feel like you have not had enough time to really grasp the procedure (Male student, 24 years)”*.

##### 3.2.4.3 Lack of high-quality manikins

The lack of high-quality manikins was another challenge frequently mentioned by both students and faculty. High-quality, realistic manikins are essential for effective simulation training, as they allow students to practice procedures and skills in a way that closely mimics real-life scenarios. However, the availability of such resources was often lacking. A student expressed this challenge, saying, “*The manikins we use are also old some of them need repair…. It can be hard to take the simulation seriously when the equipment does not behave like a real patient would (Female Student, 23 years)”*.

##### 3.2.4.4 Inadequate faculty training

A critical challenge identified was the inadequate training of faculty members in using simulation equipment and facilitating simulation-based learning. Effective simulation training requires instructors who are not only subject matter experts but also skilled in guiding students through realistic scenarios and debriefing sessions. However, many faculty members expressed concerns about their preparedness. One faculty member candidly admitted, “*I feel like we are thrown into these sessions without proper training in our first year of training…. we do not know how to operate the equipment or how to debrief the students afterward. It is a bit challenging to lead a session when you are not fully comfortable with the tools yourself (Resident, 27 years)*”.

#### 3.2.5 Suggestions for improving

The suggestions centered around four main subthemes: multi-media support, provision of high-quality manikins, punctuality for scheduled sessions, and provision of handouts.

##### 3.2.5.1 Multi-media support

Participants highlighted the need for integrating multi-media resources into simulation training. Multi-media tools, such as video tutorials and digital simulations could enhance the learning experience by providing different perspectives and reinforcing key concepts. One student remarked, *“I think having video demonstrations before the hands-on sessions would be really helpful. It gives you a visual understanding of the procedure whether it is stitching an epi…. before you actually try it yourself (Female Student, 24 years)”*.

##### 3.2.5.2 Provision of high-quality manikins

Another recurring suggestion was the need for high-quality manikins that closely mimic real-life scenarios. Participants emphasized that realistic manikins are crucial for effective simulation training, as they allow students to practice and refine their skills in a controlled, yet realistic environment. One faculty member expressed frustration with the current equipment, stating, *“The manikins we use do not always provide the level of realism that’s necessary for students to grasp the complexity of certain procedures… I think money should be spent to acquire better manikins as they would improve the quality of the training (Resident, 27 years)”*.

##### 3.2.5.3 Punctuality for scheduled sessions

Punctuality for scheduled sessions was another suggestion that came up frequently in the interviews. Both students and faculty stressed the importance of starting and ending simulation sessions on time, as delays can disrupt the learning process and reduce the overall effectiveness of the training. A student pointed out, *“When sessions start late, it throws off the whole mood… and sometimes we do not get through everything we are supposed to. It’s frustrating because we want to make the most of these opportunities (Female Student, 25 years)”*.

##### 3.2.5.4 Provision of handouts

Finally, participants suggested the provision of handouts as a way to reinforce learning and provide students with reference material that they can review after the sessions. These handouts could include key concepts, step-by-step guides, and checklists to help students retain what they have learned during the simulation. One student explained, *“Having handouts would be really helpful because sometimes it’s hard to remember everything that was covered in the session. A handout would give us something to look back on and study (Female Student, 24 years)”*.

#### 3.2.6 Triangulation of study findings

The triangulation of the study findings was also done, shown in Table 7.

**Table 7:** Triangulation of study findings.

Theme/Domain	Quantitative findings	Qualitative findings	Interpretation
Experiences during workshop sessions-workshop content	Mean improved from 13 (SD = 2.3) to 9.97 (SD = 2.1) t-value: 15.7, *p* < 0.00	“The simulation session was very structured. It followed a clear form… our clinical coordinator gave us a proper schedule.” (female student, 24 yrs)	Quantitative improvement supports qualitative experiences of clarity and structure in workshops.
Experiences during workshop sessions-resource availability	Mean improved from 12 (SD = 2.4) to 16.8 (SD = 2.9) t-value: 18.1, CI: 4.2–5.3, *p* < 0.00	“The manikins we use are also old… It can be hard to take the simulation seriously when the equipment does not behave like a real patient.” (female student, 23 yrs)	Quantitative gain aligns with calls for improved resources and tools.
Perceived technical benefits-Clinical ability	Highest gain: t-value = 24.6, CI: 7.4–8.7, *p* < 0.00	“Before the simulation sessions, I struggled with certain procedures… But after practicing… my hands just seemed to know what to do.” (male student, 22 yrs)	Strong quantitative and qualitative agreement on clinical skill development.
Perceived behavioral Proficiency-confidence	t-value: 16.0, CI: 4.3–5.5, *p* < 0.00	“Before the simulations, I was always second-guessing myself… I feel much more confident when I’m in the actual labour room.” (female student, 23 yrs)	Reinforces simulation’s role in boosting clinical confidence.
Perceived behavioral proficiency-Problem solving	t-value: 16.5, CI: 5.3–6.7, *p = 0.07*	“It’s one thing to know the steps in managing a case; it’s another to actually go through the process, make decisions, and see the outcomes.” (male student, 24 yrs)	Near-significant result supports perceived improvement in critical thinking.
Perceived behavioral proficiency-Teamwork	t-value: 10.7, CI: 2.5–3.6, *p* < 0.00	“The team-based scenarios… allowed them how to rely on their colleagues and also how to contribute effectively… they learnt to trust each other.” (consultant, 44)	Quantitative gains align with qualitative reports of collaborative learning.
Overall learning effectiveness	Pre-score: 89.4 → post-score: 139.5 Mean difference: 50.1 Learning gain ratio: 56% t = 6.7, CI: 13.6–7.4, *p* < 0.00	“Simulation is an excellent adjunct… students can make mistakes and learn from them without real-world consequences*.”* (consultant, 42 yrs)	Strong convergence showing training significantly improved learning.

## 4 Discussion

The study demonstrates that SBT significantly enhances learning effectiveness among undergraduate students in Gynaecology and Obstetrics to our knowledge it is the first mixed methods study on simulation training in Pakistan. The results of this study reveal statistically significant improvements in various aspects of student learning, echoing recent evidence that supports the positive impact of simulation-based education in medical training.

Students reported an improved perception of the workshop content after the intervention, indicating that SBT offers a more structured and comprehensive learning experience in Gynaecology and Obstetrics. This aligns with Fransen et al. who highlighted the value of well-structured simulation workshops in mastering complex medical procedures in Gynaecology and Obstetrics (7). Perceptions of resource availability improved dramatically. Evidence affirm that high-quality simulation resources are essential for accurate clinical scenario replication and effective learning outcomes (6). Enhanced debriefing quality post-intervention aligns with evidence, which found structured debriefs improve clinical reasoning and communication skills. Students reported significant increases in their clinical abilities, supported a study which noted that simulation training improves the management of complex clinical scenarios (8). Studies further corroborate the role of simulation in boosting students’ confidence in high-stakes situations (6–9). Significant enhancements were observed in categories such as Workshop Content, Resource Availability, Clinical Ability, and Confidence, underscoring the robustness of SBT. Research provide additional support for these findings (7, 11, 13).

The qualitative analysis revealed five key themes: experience during simulation sessions, perceived technical proficiency, perceived behavioral benefits, challenges in Implementation, and Suggestions for Improvement. These themes reflect the multifaceted impact of simulation training on participants. Both students and faculty emphasized the importance of a structured approach in simulation sessions, which reduces cognitive load and enhances skill acquisition. Evidence highlights similar findings (17, 18). The hands-on nature of SBT was identified as a significant advantage, supporting the development of procedural skills as noted by different studies (20–24).

Enhanced interaction between students and facilitators facilitated personalized feedback, a finding supported by Gaba and Howard. Participants recognized that SBT complements traditional teaching rather than replacing it, which is consistent with the integrative role of simulation noted in a study (11). It supplements the evidence that simulation significantly enhances motor skills and prepares students for high-pressure situations, aligning with findings (22, 23, 25–28).

Key challenges identified include high costs of simulation technology, limited availability of trained instructors, and time constraints. These barriers are documented in existing literature (9, 11–13). Participants recommended integrating multimedia resources, providing high-quality manikins, ensuring punctuality for sessions, and distributing handouts to reinforce learning. This is supported by studies highlighting the positive effects of multimedia on engagement and the importance of realistic manikins (29–31).

Prior to the implementation of this simulation-based teaching, clinical rotation in Gynaecology and Obstetrics encompassed traditional methods, including bedside teaching, didactic lectures, and observation of real-time deliveries during hospital rotations. While these methods offered exposure to real patients, they often lacked structured skill training, uniformity and safe spaces for repetitive practice, especially given limitations in case availability and student-to-patient ratios. The simulation-based format introduced in this study represented a significant pedagogical shift. The simulation sessions are now being regularly offered as a standard part of the clinical rotation, and efforts are underway to expand the simulation component to cover additional obstetric emergencies and gynaecological procedures.

### 4.1 Strengths and limitations

This study has several notable strengths. First, a mixed methods research design has strong potential to inform the researchers as it describes congruence between the quantitative analysis of effectiveness of SBT as well as in depth exploration of its practical implications in a qualitative format. Secondly the use of standardized instruments in the research increases its validity and reliability. Thirdly, this research assessed learning effectiveness in a safe and controlled environment for medical students to practice clinical skills and procedures commonly encountered in Gynaecology and Obstetrics. Lastly, the study identified significant barriers to the application of knowledge that can be addressed. However, it also has limitations. Firstly, generalizability as the findings drawn from a tertiary care setting may not be broadly applicable across different settings or populations. In the qualitative analysis, one potential limitation was the challenge of maintaining objectivity. To address this, we involved two investigators in different stages of data coding and analysis to help minimize bias. One key limitation of this study is that our findings rely exclusively on students’ self-assessment of perceived competence and confidence, rather than objective measurement of skill acquisition. While self-reported confidence is a meaningful indicator of learner engagement and perceived preparedness, it does not necessarily correlate with actual clinical performance. Another limitation was that our study lacked objective assessment methods, such as direct structured observation, checklists, or pre- and post-simulation OSCEs (Objective Structured Clinical Examinations). This was a deliberate decision based on time constraints, faculty availability, and resource limitations during implementation. However, we recognize the value of integrating standardized skills assessments in future research to more rigorously evaluate actual performance improvement. These limitations highlight the complexities involved in conducting mixed methods of research in medical education and underscore the need for careful consideration when interpreting the study findings.

### 4.2 Implications for policy and practice

The findings of this study have several important implications for policy and practice in medical education. First, the significant improvements in clinical ability, confidence, and collaboration following the simulation workshop underscore the need to institutionalize structured, simulation-based training as a core component of the Gynaecology and Obstetrics training. Policies should support the integration of simulation into routine teaching with defined learning objectives, standardized assessments, and dedicated academic credit. Second, the reported challenges such as inadequate faculty training, limited practice time, and poor-quality manikins highlight the urgent need for investment in simulation infrastructure and human resources. Institutions must allocate funding for high-fidelity equipment and establish faculty development programs to ensure instructors are equipped to facilitate and debrief effectively. Lastly, the strong alignment between theoretical knowledge and practical application observed in both the quantitative data and participant reflections suggests that simulation serves as an effective bridge between classroom learning and real-world clinical practice.

## 5 Conclusion

This study demonstrates that SBT significantly improves the learning effectiveness of undergraduate students in Gynaecology and Obstetrics. There was a significant increase in domains such as workshop content, resource availability, clinical abilities, and confidence. Participants highlighted the learning effectiveness and facilitated the transition from theoretical knowledge to practical application. However, challenges such as high costs, limited instructor availability, and logistical constraints were also highlighted.

## Data Availability

The raw data supporting the conclusions of this article will be made available by the authors, without undue reservation.
